# Adiponectin blood levels and autism spectrum disorders: a systematic review

**DOI:** 10.1186/s12888-024-05529-1

**Published:** 2024-01-31

**Authors:** Mohsan Ali, Maha Kamran, Muhammad Talha, Mujeeb U. Shad

**Affiliations:** 1 King Edward Medical University, Lahore, Pakistan; 2 University of Nevada, Las Vegas, NV USA; 3 Touro University Nevada College of Osteopathic Medicine, Las Vegas, NV USA; 4 The Valley Health System, Las Vegas, NV USA; 5 Combined Military Hospital Lahore Medical college and institute of Dentistry, Lahore, Pakistan

**Keywords:** Adiponectin, Plasma, Levels, Autism, Spectrum, Disorders

## Abstract

**Objective:**

To review the relationship between adiponectin levels and autism spectrum disorders (ASDs) in children.

**Background:**

ASDs are associated with pervasive social interaction and communication abnormalities. Researchers have studied various pathophysiological mechanisms underlying ASDs to identify predictors for an early diagnosis to optimize treatment outcomes. Immune dysfunction, perhaps mediated by a decrease in anti-inflammatory adipokine, adiponectin, along with changes in other adipokines, may play a central role in increasing the risk for ASDs. However, other factors, such as low maternal vitamin D levels, atherosclerosis, diabetes, obesity, cardio-metabolic diseases, preterm delivery, and oxytocin gene polymorphism may also contribute to increased risk for ASDs.

**Methods:**

Searches on the database; PubMed, Google Scholar, and Cochrane using keywords; adiponectin, adipokines, ASD, autism, autistic disorder, included English-language studies published till September 2022. Data were extracted on mean differences between adiponectin levels in children with and without ASDs.

**Results:**

The search yielded six studies providing data on adiponectin levels in young patients with ASDs. As can be seen from Table 1, four of the six studies were positive for an inverse correlation between ASD and adiponectin levels. In addition, two of the four positive and one negative studies found low adiponectin levels associated with and the severity of autistic symptoms. However, results from one reviewed study were insignificant.

**Conclusion:**

Most studies reviewed yielded lower adiponectin levels in children with ASDs as well as the severity of autistic symptoms.

**Supplementary Information:**

The online version contains supplementary material available at 10.1186/s12888-024-05529-1.

## Background

Autism Spectrum Disorders (ASDs) have been diagnosed in 1% of children worldwide [1], implying that 1 in every 68 children has autism, with boys having a significantly higher risk for this disorder than girls [2]. The growing number of young patients with ASDs over the recent years, with increasing levels of mental, physical, socioeconomic, and emotional stressors, not only directly impacts autistic patients [3], but also increases the caretaker burden [4, 5]. Therefore, there is an urgent need for timely diagnosis to initiate early treatment interventions to optimize long-term outcomes in patients with ASDs [6].

Researchers have studied various pathophysiological mechanisms underlying ASD to develop early predictors for identifying at-risk patients and initiating timely treatment interventions. It is considered a complex genetic disorder having diverse familial inheritance patterns and an estimated possible involvement of up to 1000 genes [7]. Epigenetic mechanisms such as DNA methylation function at the intersection of genetic, environmental, and protective factors [8]. ASD pathogenesis is centered on altered neural connections and synaptic function. For instance, Numerous of the 207 SFARI genes that are syndromic and category 1 high risk (for ASD) encode for proteins that are essential for synaptic function in the brain. Nutritional factors, such as lower maternal vitamin D levels, have also been implicated with a greater risk of ASD in the offspring [9]. In addition, congenital and maternal infections have been correlated with ASD [10,11]. However, the link between ASD and activated immune responses in mother [12], cytokine storm [13], maternal antibodies, and auto-antibodies [14] stand out. In this context, the cytokines released from the adipocytes may play a significant role in the mediation of ASDs [15]. Beyond its energy-preserving abilities, adipose tissue is often forgotten in its role as an endocrine organ that mobilizes inflammatory reactions through the production of adipokines [16]. Not only does adipose tissue control inflammation, but it also prevents metabolic disturbances and maintains homeostasis [17], which makes it worthy of attention in its association with ASDs. Like cytokines, adipokines can also be anti- and proinflammatory, such as adiponectin and tumor growth factor-beta (TGF-β) are anti-inflammatory, and tumor necrosis factor α (TNF-α), interleukin 6 (IL-6), leptin, resistin, angiotensinogen, and plasminogen activator inhibitor-1 promote inflammation [18]. An altered ratio between anti-inflammatory [19] and proinflammatory adipokines has been reported in autistic children [20]. Of these, a reduction in the most abundant anti-inflammatory adipokine, adiponectin, has been documented to play a central role in ASDs and associated disorders, such as atherosclerosis [21], diabetes [22], obesity [23], panic disorders [24], and cardio-metabolic diseases [25].

In the past, some researchers have investigated the correlation of blood levels of adipokines with ASDs. They report elevated levels of leptin, ghrelin, resistin, and visfatin, along with decreased levels of adiponectin, retinol-binding protein 4, and progranulin were found to be associated with an increased risk of or were correlated with ASD [26]. We aim to write a review that is not only more updated than the previous reviews but also has a primary focus on adiponectin. Further, this paper also discusses the probable role of adiponectin in the pathogenesis of ASDs. The review also provides a synopsis of similar data for other adipokines and biologically plausible factors underlying ASDs.

## Methods

We followed PRISMA guidelines to perform the data review. Data searches were performed on PubMed, Google Scholar, and Cochrane using the keywords; adiponectin, ASD, autism, autistic disorder, and a combination of these. The initial screening revealed (Autism Spectrum Disorder = 45,662 results, adiponectin = 22,928, and serum adiponectin = 7,440, but the focused search narrowed down to relevant articles, for example, the detailed search strategy used to get those 12 articles (on PubMed) was, “(“arthropod struct dev“[Journal] OR “agron sustain dev“[Journal] OR “asd“[All Fields] OR (“autism spectrum disorder“[MeSH Terms] OR (“autism“[All Fields] AND “spectrum“[All Fields] AND “disorder“[All Fields]) OR “autism spectrum disorder“[All Fields])) AND (“adiponectin“[MeSH Terms] OR “adiponectin“[All Fields] OR “adiponectin s“[All Fields] OR “adiponectine“[All Fields] OR “adiponectins“[All Fields] OR “GBP-28“[All Fields] OR “apM1“[All Fields] OR “AdipoQ“[All Fields] OR “Acrp30“[All Fields])”.

### Inclusion criteria

All English-language studies published with data on the relationship between blood adiponectin levels and ASDs were selected. Data were extracted as the mean difference between blood adiponectin levels in children with and without ASDs. Since the mean differences were already adjusted, no meta-regression analysis was attempted. Observational studies, including cross-sectional or prospective studies, were eligible for this review. However, case reports, case series, duplicates, and reviews were excluded.

### Sensitivity analysis

Quality assessments were conducted using the Newcastle-Ottawa Scale (NOS) [27] for the prospective studies, while the Modified Newcastle-Ottawa Quality Assessment Scale [28] was employed for the cross-sectional studies. Two reviewers independently (T, M, and A, M) conducted the analysis, and the discrepancies were sorted through discussion, till a consensus was reached.

## Results

**Fig. 1:**
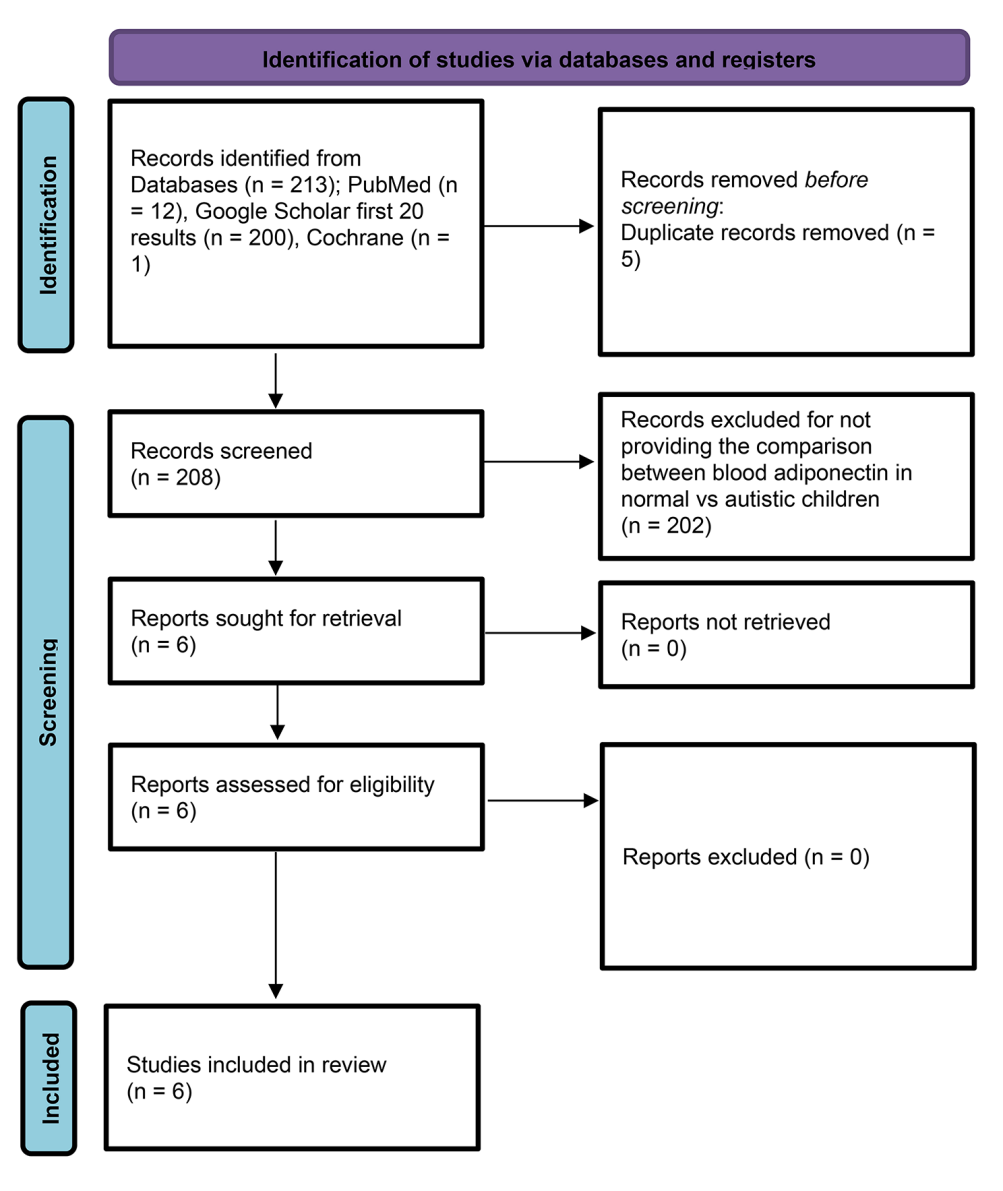
PRISMA Flow-chart

The screening and selection strategy for the studies is outlined in Fig. 1. Six studies were identified, meeting the inclusion criteria. Extracted data from these studies were pooled for analysis (Table 1). Subjects’ composition for each study is as follows: The first study [29] had a sample size of 62 males, 31 with autism, and 31 healthy age-matched controls. There was a statistically significant (*p* = 0.005) decrease in adiponectin levels in the group of males having autism (11.0 ± 4.0) as compared to healthy males (14.5 ± 5.3). In addition, an inverse association was also found between adiponectin levels and domain A scores on Autism Diagnostic Interview-Revised (ADI-R) [30]. Next study [31] compared 35 children with autism and 35 healthy controls with a one-year follow-up. No significant association was observed between ASDs or autistic symptoms and adiponectin levels. Essa et al. 2011 [32] had a sample size of 38 (19 Autistic and 19 normal children). There were significantly lower levels of adiponectin in Omani children with ASDs as compared to controls (*p* < 0.0001). The study by Rodrigues et al. [33] had a sample size of 49, (ASD = 30, Neurotypical = 19). Although no significant difference in adiponectin levels was found between neurotypical (12.91 ± 0.12) and autistic children (12.20 ± 0.38), the study did find a negative correlation between adiponectin levels and autistic scores on the Social Responsiveness Scale (SRS) [34]. Raghavan et al. 2018 [35] had a total sample of 847 [Neurotypical = 792; ASD = 55]. This study reported a significant inverse relationship between adiponectin levels and risk for ASDs. The childhood levels were only significantly correlated with risk for ASDs after controlling for age and other covariates. Quan et al. 2021 [36] compared 88 children with ASD with 88 normal children. The difference in adiponectin levels between the two groups was significant (t = 7.169, *p* < 0.001). This study also documented an inverse correlation between blood adiponectin levels and scores on Childhood Autism Rating Scale (CARS) [37] (Table 1). One of the reviewed studies [32] was of low quality, two were of moderate quality [31, 33], and three studies [29, 35, 36] were of decent quality (Table 2).

**Table 1:** Basic and clinical characteristics of study subjects in the reviewed studies

References	Age in years (mean ± SD)	Sample size	Gender (M/F)	Study design	Adiponectin Levels in µ/ml (mean ± SD range)	Adiponectin levels and ASD	Adiponectin levels and autistic symptoms
Fujita-Shimizu et al. 2010 [29]	Autism 11.6 ± 2.9 HC 12.1 ± 2.4	31 Autism; 31 HC	Autism:31/0 HC: 31/0	CS	Autism 11.0 ± 4.0 HC 14.5 ± 5.3	Inverse association between adiponectin levels and ASD (*p* < 0.005)	Inverse association between adiponectin levels and domain A scores on ADI-R
Blardi et al. 2010 [31]	Autism 14.1 ± 5.4 HC age matched	35 Autism 35 HC	Autism 21/14 HC: gender matched	PROS	Autism 10.3 ± 4.5 HC 9.9 ± 3.1	No significant differences between groups.	NA
Essa et al. 2011 [32]	Autism 3–10 years HC age matched	19 Autism 19 HC	Autism 15/4 HC: 10/9	CS	Autism (13.17 ± 0.54) HC (18.29 ± 0.65)	A significant inverse relationship between blood adiponectin and autism (*p* < 0.0001).	NA
Rodrigues et al. 2014 [33]	NA, age matched groups	30 autism 19 HC	N/A	CS	Autism (12.198 ± 3.78) HC (12.907 ± 1.22).	-No significant relationship between autism and adiponectin levels	Inverse correlation between adiponectin levels and SRS scores.
Raghavan et al. 2019 [35]	NA	ASD = 55 HC = 792	Autism 40/15 HC 337/445	PROS	-Autism (10.11 ± 73.59) HC (10.67 ± 62.15) -Cord adiponectin levels Autism 15.80 (10.84) HC 11.37 (88.27)	-Only cord adiponectin levels inversely correlated with autism risk (*p* < 0.001) -Early childhood levels significant after controlling for confounders (*p* < 0.02)	NA
Quan et al. 2021 [36]	ASD 4.3(1.2) HC 4.3(1.2)	88 ASD 88 HC	ASD: 68/20 HC: 68/20	CS	ASD (9.01 ± 2.19) HC (11.55 ± 2.32)	Inverse correlation between adiponectin levels and autism (*p* < 0.001) and severity of autism scores (*p* < 0.001)	Negative correlation between adiponectin levels and scores on the Childhood Autism Rating Scale

ASD = autism spectrum disorder; HC = healthy controls; ADI-R = Autism Diagnostic Interview—Revised; SRS = Social Responsiveness Scale; CARS = Childhood Autism Rating Scale; CS = cross-sectional; PROS = prospective

**Table 2:** Quality analysis of the reviewed studies

Cross-Sectional Studies	Modified Newcastle Ottawa Scale Score/Total Score
Fujita-Shimizu et al.(29)	9/10
Essa et al.2011(32)	4/10
Rodrigues et al. 2014(33)	8/10
Quan et al.2021(36)	9/10
**Prospective Study**	**Newcastle Ottawa Scale Score/Total Score**
Raghavan et al.2019(35)	8/9
Blardi et al.2010(31)	7/9

## Discussion

This, to our knowledge, is the first systematic review to investigate the association between adiponectin levels and the risk of ASDs. Out of six studies reviewed, four studies reported a negative correlation between autism and adiponectin levels [29, 32, 35, 36]. Lower adiponectin levels in subjects with autism than the matched controls were first reported by Fujita-Shimizu and colleagues [29] with no group differences in body weight, height, waist circumference, or BMI (Table 1). However, the next study [31] did not observe any significant differences in adiponectin levels between ASD patients and controls. But the study by Essa et al. [32] replicated the findings from the first study in Omani children and showed an inverse relationship between adiponectin levels and those with ASD. The study by Rodrigues et al. [33] was another study that did not show a negative relationship between adiponectin levels and ASD patients. The next study [35] was the first to prospectively report an inverse association between ASD risk and adiponectin levels in the blood during early childhood. However, the association between early childhood adiponectin and ASD was less robust and achieved significance only after stepwise adjustments for potential confounders. The correlation between adiponectin and ASD parallels an increase in adipose tissue that switched into a negative correlation after birth [38]. Other studies have also reported a positive relationship of adipose tissue with adiponectin [39] converting into a negative association with adiponectin in early childhood [40, 41]. The last study by Quan et al. [36] replicated findings from prior studies [29, 32], reporting lower adiponectin levels in patients with ASDs than controls.

Although Rodrigues et al. [33] did not find a negative association between adiponectin levels and ASDs in their study subjects, they did report an inverse correlation between blood adiponectin levels and severity of autistic symptoms, as assessed with the Social Responsiveness Scale (SRS) [42]. Fujita-Shimizu and Colleagues [29] also reported an inverse correlation between adiponectin levels and autistic symptoms of abnormalities in social interactions, as assessed with the Autism Diagnostic Interview-Revised (ADI-R) [30]. Interestingly, the adiponectin levels were not correlated with autistic symptoms of repetitive behaviors and restricted interests but with social [29]. These findings suggest that low adiponectin levels may not affect all autistic symptoms. Another reviewed study [36], supported an inverse correlation between adiponectin levels and symptom severity as assessed with Childhood Autism Rating Scale (CARS) [37]. However, in one of the reviewed studies, adiponectin levels were not associated with ASDs or the severity of autistic symptoms [31].

In addition to adiponectin, autism has also been associated with changes in other adipokines, such as leptin. The study by Blardi et al. [31] found higher blood levels of leptin in autistic patients than in controls over one year without any differences in adiponectin levels, which is consistent with findings from an earlier study [43]. However, gender differences in leptin levels in females [44] were not reported in the study by Blardi et al. [31]. Also, increased leptin levels were not associated with obesity, suggesting that leptin may have effects beyond adipose tissue and energy balance [31]. The inverse relationship between adiponectin and ASDs has also been reported in other neurodevelopmental disorders, such as Fragile X Syndrome [45]. These findings suggest that adiponectin may have a larger role in neurodevelopment rather than merely regulating energy expenditure or serving as a biomarker for the onset of metabolic syndrome [46, 47]. Any imbalance in adiponectin-mediated anti-inflammatory effects [48] and/or leptin-mediated proinflammatory effects [49, 50] may alter the immune response increasing the risk for ASDs. This immune dysfunction is supported by an inverse relationship between leptin and adiponectin in autistic children [33, 47]. In addition, like adiponectin, leptin can have direct brain effects as it can cross the blood–brain barrier [51]. Another adipokine involved in ASDs may be resistin, which has more potent proinflammatory effects than leptin [52]. Although one of the reviewed studies [33] reported decreased levels of resistin, other studies have reported an increase in resistin levels^20^, supporting a more enhanced inflammatory response underlying ASDs [51]. In addition to ASDs, altered levels of adipokines have also been reported in other brain disorders, such as bipolar disorder [53] and Alzheimer’s dementia [54]. These variations in adipokine levels across different neuropsychiatric disorders may reflect biological differences underlying these diseases. Further, the clinical heterogeneity observed in autism may be attributed to the diverse content in its etiology because there has been a significant correlation between the variation (decrease) in blood adiponectin levels and the severity of clinical symptoms as in the study by Quan et al. [36] where higher blood adiponectin levels were associated with milder clinical severity. This warrants further, focused, research on the neurobiology and undiscovered mechanisms, that affect clinical severity on the social responsiveness scale.

Although the biological mechanisms underlying adiponectin changes are not fully understood, prior studies have hypothesized multiple explanations. One of the most plausible explanations in the reviewed studies is provided by an inflammatory basis of ASDs [29, 32, 33, 35, 36]. In this context, the anti-inflammatory role of adiponectin in suppressing proinflammatory cytokines, such as TNF-α, IL-6 [55, 56], and Interferon (IFN)-γ [56, 57], that are elevated in ASD [56, 58, 59].

According to recent research, oxidative stress and variation in genes encoding antioxidant enzymes may have a role in the development of ASD [60]. A study showed that adiponectin leads to a decrease in mitochondrial ROS formation and oxidative DNA damage thereby lowering the odds of developing ASDs. It also improves mitochondrial dysfunction by boosting Bcl-2 levels and inhibiting the production of active caspase-3 and Bax [61]. The biological relevance of adiponectin’s role in ASD could also be explained by the disturbances in the metabolic pathways like folate, tetrahydrobiopterin, and glutathione-dependent redox metabolism that are seen in children with ASDs [62]. Adiponectin protects against inflammatory reactions linked to metabolic abnormalities (metaflammation) such as obesity or insulin resistance [57], hence playing a role against the metabolic derangements in ASDs.

Adiponectin has also been proposed to promote neuroplasticity, perhaps mediated by a similar mechanism as the ketamine-induced nitric oxide synthase signal pathway, which also mediates rapid antidepressant effects [55, 63]. Adiponectin crosses the blood-brain barrier [64], and modifies neuronal activity in several brain regions where it has neuroprotective and neurotrophic effects. Studies have shown that adiponectin can mediate its neuroprotective effects in the hippocampus [65], hypothalamus, cortex, and pituitary glands [55, 63] by entering the brain circulation [63, 66]. For example, in the hippocampus, adiponectin is involved in neurogenesis, dendritic spine remodeling, and hippocampal stem cell proliferation [65, 66]. Abnormal development of the dentate gyrus of the hippocampus is likely implicated in the pathophysiology of ASD [67]. In addition, these hippocampal changes may play an important role in maintaining mental health. A study found that an increase in adiponectin levels exerts powerful antidepressant and anxiolytic effects, particularly by fighting against neuroinflammation [68]. In this context, decreased adiponectin levels have been associated with clinically significant affective episodes [69] and increased sympathomimetic activity, as observed in depression [70]. While an increase in adiponectin has been associated with antidepressant and anxiolytic effects, perhaps due to its anti-inflammatory effects [68].

Overall, having such a broad-spectrum proforma and being the most abundant adipokine, adiponectin may contribute to metabolic and immune homeostasis via adipocyte-brain communications [63]. However, findings from this review warrant a cautious interpretation as they are based on six small sample studies. Since all reviewed studies were observational, no causality relationship can be claimed, particularly in the presence of multiple confounding factors.

## Conclusions

This review provides preliminary evidence for the ASDs and severity of autistic symptoms associated with altered levels of adipokines, particularly adiponectin. However, the inconsistent results from some studies could be explained by the biological heterogeneity across ASDs, smaller study samples, and less-than-optimal study designs. These shortcomings can be addressed by prospective longitudinal studies in larger samples with a broader age spectrum to validate the results from this review. An even better strategy would be to develop neurobiological and genetic predictors for early diagnosis and treatment response to optimize disease prognosis by using a translational neuroscience approach.

### Electronic supplementary material

Below is the link to the electronic supplementary material.

Supplementary Material 1

## Data Availability

The datasets used and/or analyzed during the current study are available from the corresponding author upon reasonable request.
